# The association between sarcopenia and bladder cancer-specific mortality and all-cause mortality after radical cystectomy: A systematic review and meta-analysis

**DOI:** 10.1080/2090598X.2021.1876289

**Published:** 2021-01-16

**Authors:** Christine Ibilibor, Sarah P. Psutka, Jesus Herrera, J. Ricardo Rivero, Hanzhang Wang, Ann M. Farrell, Michael A. Liss, Deepak Pruthi, Ahmed M. Mansour, Robert Svatek, Dharam Kaushik

**Affiliations:** a Department of Urology, University of Texas Health Science Center at San Antonio, San Antonio, TX, USA; b Department of Urology, University of Washington, Seattle, WA, USA; c Plummer Library, Mayo Clinic, Rochester, MN, USA; d Mays Cancer Center, San Antonio, TX, USA

**Keywords:** Bladder cancer, sarcopenia, cancer-specific mortality, all-cause mortality, meta-analysis

## Abstract

**Objective**: To compare cancer-specific mortality (CSM) and all-cause mortality (ACM) between patients with and without sarcopenia who underwent radical cystectomy for bladder cancer.

**Materials and methods**: We performed a systematic review and meta-analysis of original articles published from October 2010 to March 2019 evaluating the effect of sarcopenia on CSM and ACM. We extracted hazard ratios (HRs) and 95% confidence intervals (CIs) for CSM and ACM from the included studies. Heterogeneity amongst studies was measured using the *Q*-statistic and the *I*^2^ index. Meta-analysis was performed using a random-effects model if heterogeneity was high and fixed-effects models if heterogeneity was low.

**Results**: We identified 145 publications, of which five were included in the meta-analysis. These five studies represented 1447 patients of which 453 were classified as sarcopenic and 534 were non-sarcopenic. CSM and ACM were increased in sarcopenic vs non-sarcopenic patients (HR 1.64, 95% CI 1.30–2.08, *P* < 0.01 and HR 1.41, 95% CI 1.22–1.62, *P* < 0.01, respectively).

**Conclusions**: Sarcopenia is significantly associated with increased CSM and ACM in bladder cancer. Identifying patients with sarcopenia will augment preoperative counselling and planning. Further studies are required to evaluate targeted interventions in patients with sarcopenia to improve clinical outcomes.

**Abbreviations:** ACM: all-cause mortality; ASA: American Association of Anesthesiologists; BMI: body mass index; CCI: Charlson Comorbidity Index; CSM: cancer-specific mortality; CSS: cancer-specific survival; ECOG: Eastern Cooperative Oncology Group; HR: hazard ratio; NAC: neoadjuvant chemotherapy; NIH: National Institutes of Health; OS: overall survival; RC: radical cystectomy; RCT: randomised controlled trial; SMI: Skeletal Muscle Index

## Introduction

In 2020, there were ~81 400 new bladder cancer cases and 17 980 bladder cancer-related deaths in the United States alone [1]. The incidence and prevalence of bladder cancer begins to increase in the sixth decade of life and peaks in the seventh to eighth decade, with a higher proportion of males affected than females [2]. For localised disease, the cornerstone of treatment remains radical cystectomy (RC), pelvic lymphadenectomy, followed by urinary diversion [3]. Despite increased utilisation of neoadjuvant chemotherapy (NAC) [4], employment of standardised enhanced recovery after surgery (ERAS) protocols [5] and incorporation of minimally invasive surgical approaches [6], perioperative outcomes, treatment-related morbidity and post-RC survival remain relatively stable [7]. Even in high-volume centres, post-RC complications occur in 25–80% of cases, with major complications occurring in up to one-third of cases. The 90-day mortality ranges between 12.8% and 14.8% in elderly patients and death within index hospitalisation of 2–3% [8,9]. After RC, 16% of patients require transitional care placement and nearly 40% of those discharged to a transitional facility postoperatively require re-admission [10].

A proposed rationale for these outcomes has been that patients with bladder cancer represent a highly comorbid population, given their age, but also a generally high prevalence of significant comorbidities, smoking, and poor performance status [11]. In the past, tools such as the Charlson Comorbidity Index (CCI), the American Association of Anesthesiologists (ASA) score, Eastern Cooperative Oncology Group (ECOG) performance status and the body mass index (BMI) have provided an incomplete assessment of the risk of morbidity and mortality after RC amongst high-risk patients [12]. In recent years, there has been increasing interest in surrogates of frailty as a potential objective risk factor. Measurement of the Skeletal Muscle Index (SMI) has emerged as a potential tool to identify deficient levels of lean muscularity, which has been shown to have associations with increased risks of complications and mortality in patients undergoing RC [13–15].

Sarcopenia refers to a severe deficiency of skeletal muscle tissue and it has been classified in accordance with international consensus definitions based on the SMI [16]. These thresholds are sex-specific and can be reproducibly measured on CT [13,16]. Furthermore, measurement of lean muscularity rely on CT at the level of the third-lumbar vertebrae has been validated against dual-energy X-ray absorptiometry (DEXA) scans and is able to estimate body composition accurately [17].

The purpose of the present meta-analysis was to aggregate all the available data regarding sarcopenia to assess the association between sarcopenia and bladder cancer-specific mortality (CSM) and all-cause mortality (ACM) in patients treated with RC.

## Materials and methods

We performed this meta-analysis in accordance with the Preferred Reporting Items for Systematic Reviews and Meta-Analyses (PRISMA) Statement [18]. An experienced librarian (A.F.) conducted a comprehensive search of the major databases (Ovid MEDLINE, Ovid EMBASE, Ovid Centre for Reviews and Dissemination, Web Science, American College of Physicians Journal Club, Database of Abstracts of Review of Effect and Scopus) with input from the study team using various keywords. The search included articles published between October 2010 and March 2019. Controlled vocabulary was used to search for randomised controlled trials (RCTs) and cohort studies comparing outcomes of sarcopenic vs non-sarcopenic patients undergoing RC for bladder cancer.

The population of interest included patients with sarcopenia undergoing RC irrespective of chemotherapy status. The comparison group included patients that were not considered sarcopenic prior to RC. To be included in the meta-analysis, studies had to be observational or population-based, report either CSM or ASM or both, report their definition of sarcopenia and mode of measurement.

The studies were independently screened by three reviewers (J.R.R., H.W., D.K.). First, titles of the retrieved articles were screened for inclusion. Next, the abstracts of the remaining articles were reviewed. We excluded studies that were single case reports, not in the English language, did not involve treatment of bladder cancer with RC, or did not have clear measurement or definition of sarcopenia. After excluding articles based on title and abstracts, a full-text review was performed on the remaining articles. Any disagreements regarding inclusion of selected articles were resolved by consensus. A data collection sheet was designed and used to collect all outcome data and study characteristics. The quality of each included study was assessed by using the National Institutes of Health (NIH) Quality Assessment Tool for Observational Cohort and Cross-Sectional Studies and were given a rating of either ‘good’, ‘fair’ or ‘poor’ [19].

Our primary outcomes of interest, CSM and ACM, were calculated by extracting and combining hazard ratio (HR) point estimates along with 95% CIs from the papers that were eligible for inclusion in the meta-analysis. The results of each study were weighted by the inverse of the variance for each outcome separately. The effect estimate was calculated using fixed effects and DerSimonian and Laird random-effects models. Heterogeneity was assessed across the studies using the *Q*-statistic and the *I*^2^ index. As a tentative benchmark, an *I*^2^ index of <40% was considered as low heterogeneity. Meta-analysis was performed using a random-effects model if statistically significant moderate or high heterogeneity was noted. Fixed-effect models were used only if low heterogeneity was present. We plotted funnel plots and used the Egger’s test to examine potential publication bias. The Begg’s rank correlation test was used to examine the association between effect estimates and their variances. Statistical software STATA (version 12; StataCorp, College Station, TX, USA) was used for all analyses.

## Results

**Figure 1.**
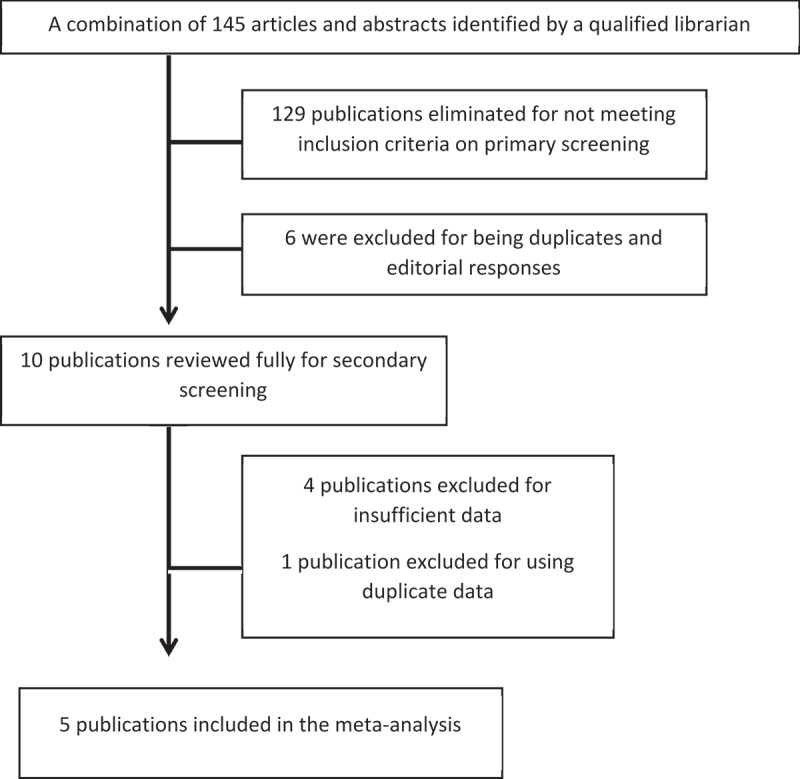
Flow chart for study selection

In total, 145 publications were screened for inclusion and 10 were selected for further review. Of these 10, five publications met the inclusion criteria and were included in the meta-analysis [20–24]. Further details of the selection criteria are shown in Figure 1. The studies included were well designed retrospective cohort studies.

The quantitative analysis included data from 1447 patients, of whom 453 were classified as sarcopenic and 534 as non-sarcopenic. The discrepancy in the total number of patients is due to the data regarding the number of sarcopenic and non-sarcopenic patients in the Harraz et al. [20] abstract being absent. The baseline characteristics for each included study and patient are given in Table 1 [20–24]. The median follow-up across the included studies was 40.8 months and included patients were largely similar with regard to median age, tumour stage, and nodal involvement. Four of the five studies used CT to determine the cross-sectional area of skeletal muscle at the level of the third lumbar vertebrae [21,22,24,25] to define sarcopenia, while Harraz et al. [20] used total psoas area (TPA).

**Table 1.** Baseline characteristics of included studies and patients

Study	Psutka et al., 2015 [24]			Harraz et al., 2015 [20]		Hirasawa et al., 2016 [21]			Miyake et al., 2017 [23]			Mayr et al., 2018 [22]	
Setting	Single centre/USA			Single centre/Egypt		Multicentre/Japan			Single centre/Japan			Multicentre/Europe	
Study type	Retrospective cohort study			Retrospective cohort study		Retrospective cohort study			Retrospective cohort study			Retrospective cohort study	
NIH quality rating	Good			Fair		Good			Good			Good	
Time period	2000–2008			2004–2008		2003–2015			2006–2016			2004–2014	
Patients, *n*	262			460		136			89			500	
Cohort comparison	Sarcopenic (S)-obese(O)/S-non-obese (NO) and non-sarcopenic (NS)-O/NS-NO			S/NS		S/NS			S/NS			S/NS	
Follow-up	6.3 years** (IQR 5.7–9.5 years)			NA		46.7 months*			29 months** (IQR 10–60 months)			35 months** (IQR 20–58 months)	
Inclusion criteria	UC treated with RC for MIBC			UC treated with RC for MIBC		UC treated with RC for MIBC			UC treated with RC for MIBC			UC treated with RC for MIBC	
Measurement used for defining sarcopenia	The cross-sectional skeletal muscle and adipose areas at the level of the third lumbar vertebra. SMI <55 cm^2^/m^2^ (men), <39 cm^2^/m^2^ (women)			TPA ≤53.5cm^2^/m^2^ (men), ≤37.4 cm^2^/m^2^ (women)		The cross-sectional skeletal muscle area at the level of the third lumbar vertebra. SMI <43 cm^2^/m^2^ with BMI <25 kg/m^2^, or SMI <53 cm^2^/m^2^ with BMI >25 kg/m^2^ (men) and SMI <41 cm^2^/m^2^ (women)			The cross-sectional area of skeletal muscle at the level of the third lumbar vertebra. SMI <43 cm^2^/m^2^ with a BMI <25 kg/m^2^, or <53 cm^2^/m^2^ with a BMI >25 kg/m2 (men) and <41 cm^2^/m^2^ for (women)			The cross-sectional skeletal muscle surface at the level of the third lumbar vertebra. SMI of <43 cm^2^/m^2^ with BMI <25 kg/m^2^ or, SMI <53 cm^2^/m^2^ with BMI ≥25 kg/m^2^ (men), and SMI <41 cm^2^/m^2^ (women)	
Primary endpoints	ACM			5-year CSS, 5-year ACM		CSS			CSS, ACM			5-year CSS, 5-year ACM	
Group	NS-NO NS-O	S-NO S-O	NS		S		NS	S		NS	S	NS	S
Patients, *n*	27 58	103 74	460				71	65		67	22	311	189
Median age, years	61 68 72 72		NA NA				71.6*	65.8*		72	71	70	73
Men, *n* (%)	13 (48) 54 (93) 86 (83) 72 (97)		NA NA				NA	NA		55 (82)	15 (68)	255 (82)	146 (77)
Median BMI, kg/m^2^	26.7 32.7 24.4 29.5		NA NA				NA	NA		23.3	21.8	27.0	26.0
History of systemic NAC, %	14.8 6.9 14.7 9.6		NA NA				4.2	6.2		43	36	NA‡	NA‡
Pathological Stage T0–T2, *n* (%)	47 (55.2) †	96 (54.2)†	NA NA				53 (74.6)^	40 (62.5)^		38 (57)	10 (45)	174 (55.9)	102 (54)
Pathological Stage T3–T4, *n* (%)	38 (44.7)†	80 (45.1)†	NA NA				18 (25.3)^	25 (38.5)^		29 (43)	12 (55)	137 (44.1)	87 (46)
Nodes positive, *n* (%)	17 (20)†	35 (39.2)†	NA NA				NA	NA		8 (12)	3 (14)	83 (23.4)	43 (22.8)

IQR: interquartile range; MIBC: muscle invasive bladder cancer; NA: not available, NO: non-obese; NS: non-sarcopenic; O: obese; S: sarcopenic; TPA: total psoas area; UC: urothelial carcinoma. *Mean; **median; †data available for 261/262 patients; ^ Clinical Stage; ‡Patients with history of prior neoadjuvant chemotherapy were excluded from the Mayr et al. [22] cohort. Harraz et al. [20] did not provide baseline characteristics in the provided abstract.

**Figure 2.**
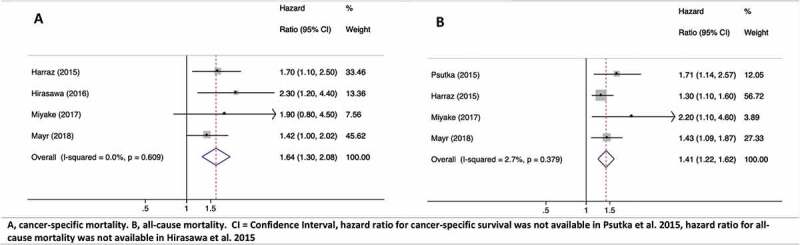
HR plots for CSM (**A**) and ACM (**B**) in sarcopenic patients. HR for CSS was not available in Psutka et al. 2015 [24] and HR for ACM was not available in Hirasawa et al. 2015 [21]

The HR for CSM was available in four of the five studies [20–23]. On multivariate analysis, there was an increased risk of CSM among sarcopenic compared to non-sarcopenic patients (HR 1.64, 95% CI 1.3–2.08; *P* < 0.01). Heterogeneity amongst these studies was found to be low (*I*^2^ = 0%, *P* = 0.609; Figure 2 [21,24]).

Similarly, the HR for ACM was available in four of the five studies [20,22–24]. On multivariate analysis, patients who were sarcopenic showed a significant increase in ACM compared to non-sarcopenic patients (HR 1.41, 95% CI 1.22–1.62; *P* < 0.01). Heterogeneity amongst the studies was also found to be low (*I*^2^ = 2.7%, *P* = 0.379; Figure 2). There was no significant publication bias for ACM and CSM (all *P* > 0.05 for the Begg’s and Egger’s tests).

## Discussion

In the present meta-analysis, we compared CSM and ACM between sarcopenic and non-sarcopenic patients who underwent RC for bladder cancer. The comprehensive review of five publications including 1447 patients indicated an increase in CSM and ACM for patients with sarcopenia when compared to those without sarcopenia. Each of the five studies included in this meta-analysis supported the role of sarcopenia as a prognostic tool for both CSM and ACM in patients undergoing RC for bladder cancer [20–24]. The Psutka et al. [13] study was one of the first to show that sarcopenic patients undergoing RC for bladder cancer had worse overall survival (OS) and cancer-specific survival (CSS) compared to non-sarcopenic patients (39% vs 70% and 49% vs 72%, respectively), thus, identifying preoperative sarcopenia as an important comorbid condition with a significant contribution to postoperative mortality in the RC population.

Hu et al. [26] performed a similar systematic review and meta-analysis comparing CSM and ACM between sarcopenic and non-sarcopenic patients with urothelial carcinoma. They included 12 studies with 2075 patients of which, five included patients with upper tract urothelial carcinoma. Of the remaining seven articles including patients with urothelial carcinoma of the bladder, two articles contain duplicated patient populations [13,24]. In their study, they found sarcopenia to be associated with diminished OS and CSS with HRs of 1.87 and 1.98, respectively [26]. Although Hu et al. [26] had similar outcomes, our present study focussed on bladder cancer avoiding upper tract urothelial carcinoma and eliminating duplicated study populations, which makes our results more robust. The Psutka et al. [13] 2014 cohort included 205 patients; however, they revisit this cohort in 2015 adding 57 patients to further explore the association between obesity and OS after RC while adjusting for the presence of lean muscle wasting [24]. Hence, although the 2014 cohort met our criteria, we excluded this study to avoid duplicating patients; however, both studies [13,24] are included in the Hu et al. [26] study. In a similar fashion, the Miyake et al. [23] 2017 study and its later version [25] included similar patient populations; however, we decided to exclude the later version given that only 23 patients where considered sarcopenic [25] vs 22 in its earlier version [23]. Although that study was not included in our meta-analysis, it is interesting to note that sarcopenia was an independent factor for OS [25], which is consistent with similar findings of its earlier version [23].

Over the last decade an emphasis has been placed on malnutrition as an unrecognised comorbid condition in patients undergoing RC for bladder cancer [11,17,23,25]. Psutka et al. [13] illustrated the poor overall health condition of patients with urothelial cancer by demonstrating in their cohort that these patients had an elevated CCI, ASA scores and worse ECOG performance status. In the Psutka et al. [13] cohort of 205 patients, 141 were classified as sarcopenic despite the median BMI of the cohort being 27.1 kg/m^2^, which is classified as overweight by the WHO. This is clinically relevant as sarcopenia can be overlooked in overweight and obese patients [27]. However, based on the baseline information of the studies included in this meta-analysis, we observed that sarcopenic patients had a relatively lower BMI compared to non-sarcopenic patients. Clinicians can use BMI changes to assist in the early detection of sarcopenia. Evidence has shown that resistance exercises are better for improving muscle strength and volume in patients with cancer [28]. Also, a RCT has shown that a preoperative exercise programme in conjunction with nutritional supplementation is feasible and can improve muscle power prior to RC as a means of preventing sarcopenia in the preoperative period and mitigating its negative effects on postoperative outcomes [28,29].

To our knowledge, this is the largest comparison to date on the effect of sarcopenia and oncological outcomes in patients undergoing RC for bladder cancer. However, the present review is not without limitations. The definition of sarcopenia varied between studies and there is currently no international consensus on its definition. All the studies included in the present systematic review and meta-analysis were considered to be of good to fair quality; however, due to their retrospective nature they are inherently prone to multiple types of biases. NAC and adjuvant chemotherapy are also important factors that affect the prognosis of patients with bladder cancer; however, there was not enough detailed data for a subgroup analysis.

## Conclusion

Sarcopenia may be an unrecognised comorbid condition and is independently associated with increased risk of ACM and CSM after RC for bladder cancer. Identifying patients with sarcopenia will help tailor patient counselling and assist in preoperative planning. Further investigation is needed to determine the extent of any intervention that may help improve outcomes of sarcopenic patients undergoing RC.
